# Persistence of the invasive bird-parasitic fly *Philornis downsi* over the host interbreeding period in the Galapagos Islands

**DOI:** 10.1038/s41598-022-06208-5

**Published:** 2022-02-11

**Authors:** Mariana Bulgarella, M. Piedad Lincango, Paola F. Lahuatte, Jonathan D. Oliver, Andrea Cahuana, Ismael E. Ramírez, Roxanne Sage, Alyssa J. Colwitz, Deborah A. Freund, James R. Miksanek, Roger D. Moon, Charlotte E. Causton, George E. Heimpel

**Affiliations:** 1 Department of Entomology, University of Minnesota, St. Paul, MN USA; 2 Charles Darwin Research Station, Charles Darwin Foundation, Puerto Ayora, Santa Cruz Island, Galapagos Islands Ecuador; 3 Facultad de Ciencias Agrícolas, Universidad Central del Ecuador, Quito, Ecuador; 4 Biology Department, University of Wisconsin Eau Claire, Eau Claire, WI USA; 5 Present Address: School of Biological Sciences, Victoria University of Wellington, Wellington, New Zealand

**Keywords:** Entomology, Invasive species

## Abstract

Many parasites of seasonally available hosts must persist through times of the year when hosts are unavailable. In tropical environments, host availability is often linked to rainfall, and adaptations of parasites to dry periods remain understudied. The bird-parasitic fly *Philornis downsi* has invaded the Galapagos Islands and is causing high mortality of Darwin’s finches and other bird species, and the mechanisms by which it was able to invade the islands are of great interest to conservationists. In the dry lowlands, this fly persists over a seven-month cool season when availability of hosts is very limited. We tested the hypothesis that adult flies could survive from one bird-breeding season until the next by using a pterin-based age-grading method to estimate the age of *P. downsi* captured during and between bird-breeding seasons. This study showed that significantly older flies were present towards the end of the cool season, with ~ 5% of captured females exhibiting estimated ages greater than seven months. However, younger flies also occurred during the cool season suggesting that some fly reproduction occurs when host availability is low. We discuss the possible ecological mechanisms that could allow for such a mixed strategy.

## Introduction

A common challenge faced by parasites is the seasonal availability of their hosts. In tropical and subtropical habitats, host supply can be limited by seasonally resource-poor conditions. In particular, plant growth can be severely restricted during tropical dry seasons, depriving herbivorous insects of food^1^. In turn, predators that depend on these insects as a food base are deprived of prey. Most species of passerine birds, for example, feed arthropods to their nestlings, which are scarce during tropical dry periods, resulting in nesting activities coinciding with rain^2–6^. This seasonality presents challenges for parasites of these birds, and we focus here on dipteran nest parasites, which have a free-living adult stage and lay their eggs in bird nests, where their larvae feed on nestlings. Many species of insects have evolved mechanisms such as seasonal arrest (diapause) or opportunistic inactivity (quiescence) to persist through times of low host availability^7^. Other species remain active while hosts are not available, some of them entering a state of reproductive diapause until hosts become available, e.g.^8–10^.

Dipteran nest parasites can cope with seasonal low host availability using one or more of three main strategies. First, pre-adult life stages may enter diapause during the host non-breeding season in old nests or off-nest sites. For example, after several months of winter diapause as pupae in host nests, adults of the Palearctic fly *Carnus hemapterus* emerge when the host nestlings hatch^11^. This fly can remain within host nests for several years in a state of prolonged diapause^12,13^. A second strategy involves long-lived adults persisting through the unfavourable season(s). In North American *Protocalliphora* nest flies, both sexes are thought to overwinter as adults^14^. Lastly, parasites may exploit host birds that are able to breed outside of the main bird-breeding season.

The avian vampire fly, *Philornis downsi*, is a Neotropical muscid fly found in mainland South America and Trinidad^15–19^ that was unintentionally introduced to the Galapagos Islands. In Galapagos it parasitizes many species of land birds, feeding on nestlings and causing high rates of mortality^20–22^. *Philornis downsi* has persisted in Galapagos since at least the 1960s and is known to attack 21 bird species (including 11 of 17 species of Darwin’s finches) from most habitats on at least 15 islands^21,23,24^. This is despite the fact that almost all known hosts in Galapagos exhibit a distinct breeding season during the hot period (January through May), which is associated with sporadic rain showers and an increase in insect food for developing nestlings^25,26^. Outside of these months (i.e. during the cool season), and especially in the dry lowland habitats, nesting by bird species known to be hosts of *P. downsi* appears to be very limited^26^. From hereon we refer to this period as the host interbreeding period. Determining how *P. downsi* individuals persist between bird breeding seasons could help to understand the success of this invasive species as well as inform efforts to manage these parasites and thus enhance bird conservation in Galapagos^21,27^.

A recent trapping study on Santa Cruz Island, Galapagos, showed that gravid, inseminated female *P. downsi* were active during and between the bird-breeding seasons, but males to a much lesser extent^28^. This study thus suggested that *P. downsi* does not persist during the host interbreeding period solely through pupal diapause. We propose two additional hypotheses to explain fly persistence: (i) females that emerge during a bird-breeding season could persist until the next breeding season, and (ii) the flies could continue to reproduce on the few bird species that do indeed breed (albeit at low levels) during the host interbreeding period. We distinguish between these hypotheses by assessing the chronological and physiological ages of wild flies captured during the host interbreeding period^29^. If adult *P. downsi* persist through the host interbreeding period, and there is no recruitment of new adults, then flies captured during that season would become progressively older. In contrast, if fly reproduction continues during the host interbreeding period, then young flies would be present throughout the host interbreeding period.

In the present study, we combined pupal dissections with a biochemical method for age estimation to establish whether *P. downsi* survive the host interbreeding period as diapausing pupae or adults. We also assessed egg loads and mating status in *P. downsi* to determine whether females that do persist over the host interbreeding period do so in a state of reproductive diapause.

## Methods

### Puparial dissections for diapause determination

We monitored *P. downsi* puparia from the nests of 15 bird species collected at nine sites on Santa Cruz Island, and one site on Isabela Island, Galapagos between January and June of the years 2017 to 2020 using methods previously described^28^. In all, 161 nests were collected containing 5745 *P. downsi* puparia. Of these, 4325 were placed individually into muslin-covered plastic cups (4.5 cm diam × 4.5 cm height) in the Insect Containment Facility at the Charles Darwin Research Station (CDRS) in Puerto Ayora, Santa Cruz Island, Galapagos at 27 ± 5 °C, 65 ± 5% R.H. and ambient photoperiod. The puparia were monitored for fly emergence, which occurs ~ 10 days after pupation under these conditions^30^. Puparia not emerging within 15 or more days of pupation were dissected to determine whether the pupae within were alive, which would be consistent with diapause. If the contents were dry, they represented dead tissue. If the contents contained moisture but were uniformly grey in colour, then the individual larva, pupa or adult within the puparia was characterized as dead if flaccid when touched with a probe. Emergence of parasitoid wasps from a small minority of puparia before dissection was noted.

### Age grading

#### Laboratory calibration of pterin accumulation with age

A pilot study showed that pterin accumulates at the same rate in *P. downsi* kept under laboratory and field-cage conditions in Galapagos (Supplementary Appendix S1), so the age calibration studies were done under laboratory conditions.

Nests of local bird species (*Camarhynchus parvulus*, *Certhidea olivacea*, *Geospiza fortis*, *G. fuliginosa*, *Mimus parvulus*) containing *P. downsi* puparia were collected between 4 June and 3 July, 2015 at two field sites on Santa Cruz Island and brought to the Insect Containment Facility for the calibration study. The sites were El Barranco (0.738418° S, 90.301670° W; 15 m asl) and Los Gemelos (0.626111° S, 90.386111° W; 500–600 m asl). Puparia were removed from nests and placed into 6 ml plastic cups. Emerging adults were placed into groups of up to five same-aged flies into clear plastic tubs (10 cm diam × 6 cm height) that were vented with mesh and supplied with a standardized papaya diet and water *ad libitum*^30^. At ages ranging from 0 to 285 days, flies were sacrificed for pterin measurement by freezing. Sample sizes and ages were *n* = 46 females between 0 and 285 days old, and *n* = 22 males between 1 and 260 days old. Heads were removed and placed with a piece of desiccant (Hammond Drierite, USA) into individual centrifuge tubes wrapped with aluminium foil and shipped dry to the University of Minnesota for analyses.

Heads were stored at − 80° C until pterin extraction, and all procedures took place in a darkened room under red light (wavelength ~ 675 nm)^31–33^. Flies were sexed based on eye characteristics, and head width was measured to the nearest 1 µm using Leica LAS EZ (Switzerland) software (Supplementary Appendix S1). To extract pterins, individual heads were homogenized in 1.5 ml microcentrifuge tubes with 50 µl of 50 mM pH 8.6 Tris–HCl buffer. An additional 950 µl of buffer was added, and the mixture was centrifuged at 10,000 rpm for 5 min. Fifty µl of the supernatant was then diluted into 150 µl 50 mM Tris–HCl buffer and transferred to one well of a sterile, clear, flat-bottom, 96-well plate (FALCON, Corning Life Sci., USA). Aliquots from 93 other heads were added to each plate, and 200 µL each of three standards were added. Standards were deionized water, 10 µg/ml of 2-amino-6,7 diphenyl-4-pteridinol (Sigma Aldrich, USA) in 1 M NaOH buffer, and 50 mM of pH 8.6 Tris–HCl buffer alone. Pterin concentration was determined using a Synergy H1 Hybrid plate reader and Gen5 v1.11 software (BioTek, USA). Relative fluorescence endpoint readings were taken using an excitation wavelength of 360 nm, emission wavelength of 450 nm, and integration set at 1 s. These settings were found to be optimal in earlier studies of muscid flies, e.g.^32^.

#### Age estimation of wild *P. downsi*

*Philornis downsi* adults of unknown age (*n* = 238 females and 143 males) were collected at El Barranco site near the CDRS in McPhail traps at weekly intervals from February 2015 through March 2016 as previously described^28^. Traps containing a blended papaya-based bait were set up in mornings and checked several times/day to recover flies before they died. Live flies were brought to the CDRS where they were freeze-killed and decapitated for head and pterin measurements as described above.

El Barranco is a lowland site that typically receives very little precipitation between June and December and sporadic rainfall between January and May^28,34^. The 2015/2016 season was considered an El Niño year^35,36^, marked by increased precipitation and land bird nesting^34,37^. We present precipitation patterns at our field site in the Supplementary Appendix S2.

### Reproductive and mating status of wild female P. downsi

We dissected the abdomens of 152 of the 238 field-caught female *P. downsi*, collected from September 2015 through March 2016 for pterin assessment, to assess their egg loads and mating status. Egg loads were determined by counting the number of mature and immature eggs present within ovaries. Mature *P. downsi* eggs were identified by brown coloration and characteristic sculpturing^38^, whereas immature eggs were fully-formed, but white and smooth. Mating status of female *P. downsi* was determined by isolating each of the three spermathecae present at the median oviducts and squashing them in Ringer’s solution between a microscope slide and cover slip for viewing with a compound microscope at 100 × magnification for visible sperm. A female was determined to be mated if sperm was present in any of her spermathecae.

### Statistical analyses

For the age-grading calibration study, we used a generalized linear model (GLM) with gamma error distribution and identity link to assess the effects of *P. downsi* sex, age, head width and the interaction between sex and head width on fluorescence using the *glm* function in R^39^. We then regressed pterin relative fluorescence separately for female and male flies on their known ages. We also investigated various non-linear fits between relative fluorescence and age but decided that using a non-linear relationship for calibration was not warranted (Supplementary Appendix S3). We used generalized additive modeling (GAM) with a cubic-regression smoothing function to assess the effect of time coded as months from February 2015 through March 2016 with samples grouped monthly on fluorescence using the *gam* function in the *mgcv* package of R^40^.

We used 2nd-order polynomial regression on the number of days since January 1 to assess the seasonality of mature and immature egg loads. Head width was included as a covariate in this model to correct for effects of body size on egg loads, which were also explored separately. We also used simple linear regression to determine the correlation between pterin relative fluorescence and mature and immature egg loads and logistic regression to test for effects of date and date^2^ (corrected for head width) on whether females were mated. Lastly, we used logistic regression to determine if probability of being mated changed with estimated values of female chronological age.

## Results

### Puparial dissections for diapause determination

Of 5745 puparia found in nests and excluding 501 puparia damaged through human contact, 4242 (80.9%) emerged as adult flies either prior to collection of the nest or in the laboratory, and parasitoid wasps emerged from 43 (0.81%). Of the puparia that did not eclose (959), 924 were dissected and contained either undistinguishable remnants of *P. downsi* larvae or pupae (48.6%) or adults (51.4%), either in formation or fully formed. None of these were alive upon dissection and thus we found no evidence for diapause in this group of *P. downsi* puparia.

### Age grading

#### Laboratory calibration of pterin accumulation with age

**Figure 1:**
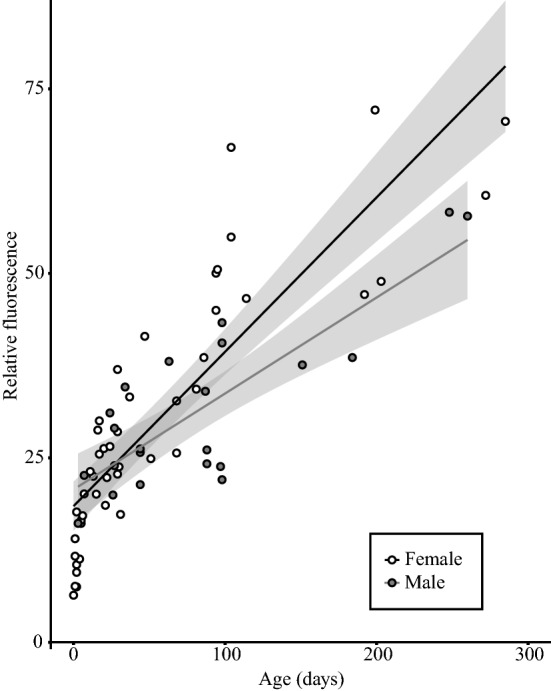
Pterin relative fluorescence (in thousands) of female and male *P. downsi* adults held under laboratory conditions until death at specified ages. Linear regression for females: Relative fluorescence = 0.21*Age + 18.46; *r*^2^ = 0.7462; *p* < 0.001. Linear regression for males: Relative fluorescence = 0.13*Age + 20.71; *r*^2^ = 0.6741; *p* < 0.001.

The GLM including sex, age, head width as well as the interaction between sex and age indicated that all of these variables had a significant effect on pterin relative fluorescence (Supplementary Appendix S3). Simple linear regressions of pterin relative fluorescence on the known ages of the 46 female and 22 male *P. downsi* held in the laboratory produced significant positive relationships with *r*^2^ values of 0.746 and 0.674 for female and male flies, respectively (Fig. 1). These linear fits were used to estimate the age of field-caught flies from relative fluorescence, leading to the following formulae:

$$\begin{aligned} & {\text{Females}}:{\text{Estimated}}\;{\text{Age}}\;\left( {{\text{in}}\;{\text{days}}} \right) = \left( {RF{-}18.45689} \right)/0.20918,\;{\text{and}} \\ & {\text{Males}}:{\text{Estimated}}\;{\text{Age}}\;\left( {{\text{in}}\;{\text{days}}} \right) = \left( {RF{-} \, 20.71339} \right)/0.13008, \\ \end{aligned}$$

where *RF* is pterin relative fluorescence (in thousands).

#### Age estimation of wild *P. downsi*

**Figure 2:**
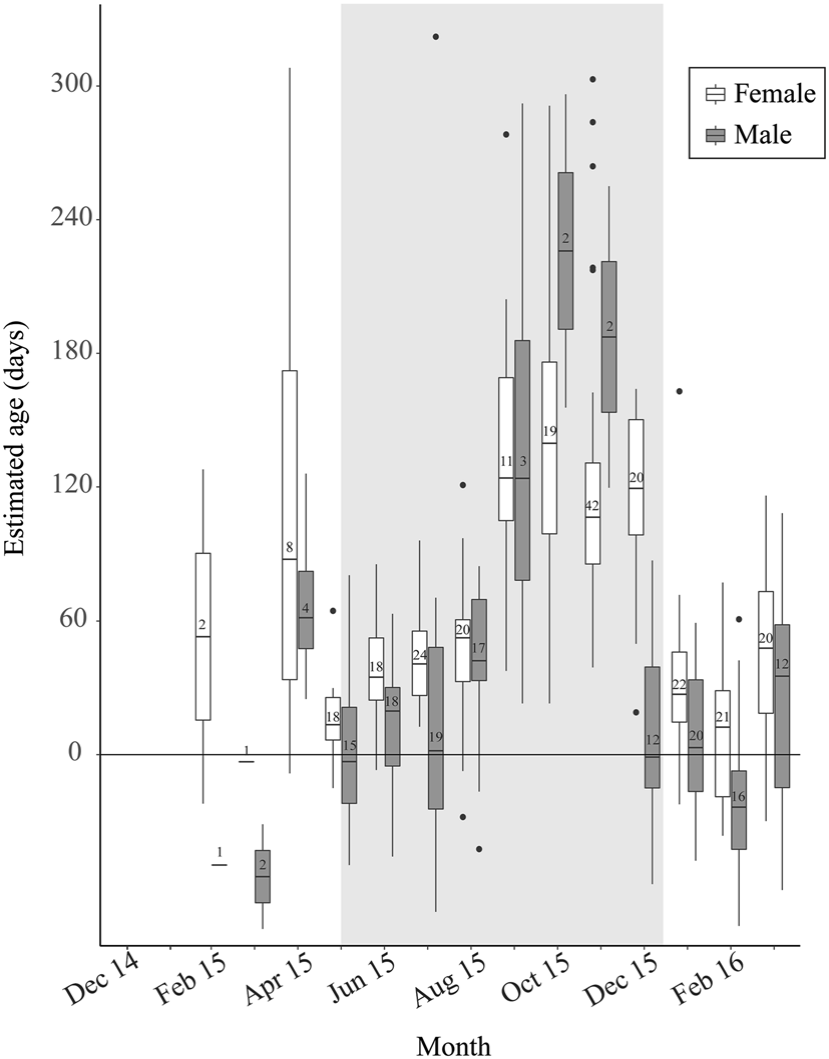
Estimated ages (in days) of adult female and male *P. downsi* flies captured at a lowland site on Santa Cruz Island, Galapagos between February 2015 and March 2016. Box plots show medians, upper and lower 75% quantiles and outliers. Numbers indicate sample size (number of flies) and the grey box indicates months that typically do not support nesting of most *P. downsi* hosts.

**Figure 3:**
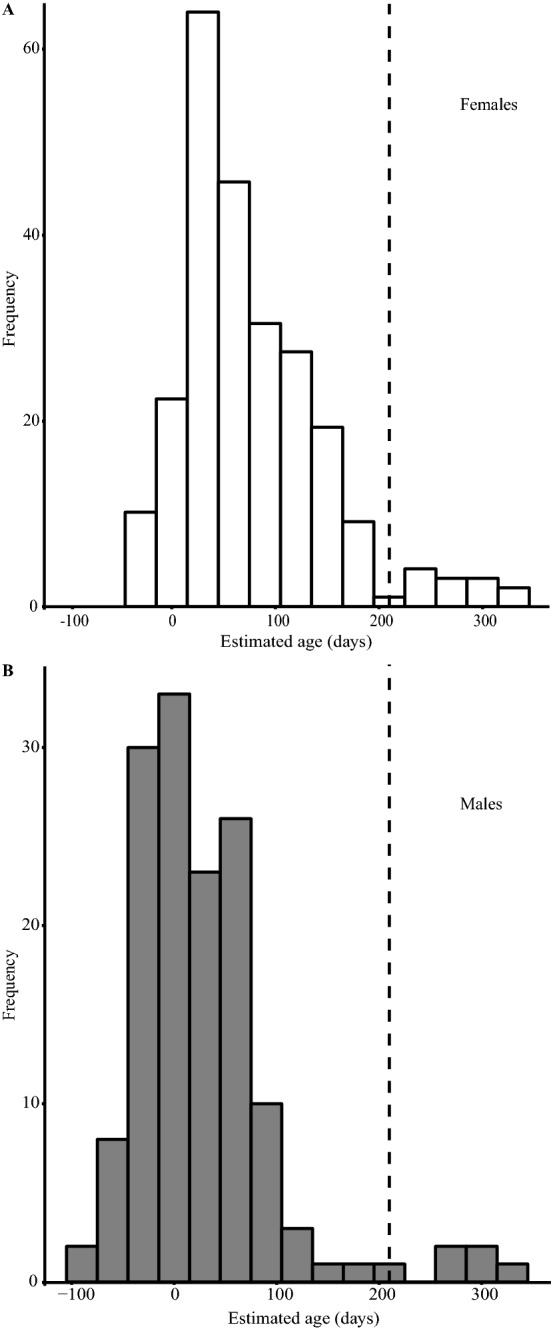
Frequency distributions of estimated ages of female (*n* = 238; **A**) and male (*n* = 143; **B**) *P. downsi* flies captured at a lowland site on Santa Cruz Island, Galapagos from February 2015 through March 2016. The vertical dotted line marks 210 days, which corresponds to the length of a typical interbreeding season, during which host availability for *P. downsi* is low.

We captured 381 adult *P. downsi* (238 females and 143 males) between February 2015 and March 2016. Maximum estimated ages were 324 and 338 days for females and males, respectively, but the average estimated age of females and males was 76.6 ± 4.5 [SE] and 27.9 ± 6.0 days, respectively. Very few males were captured during the latter half of the interbreeding season (see sample size designations in Fig. 2), suggesting that they are less capable of surviving to the end of the host interbreeding period than are females. Median estimated ages of flies ranged between 0 and 60 days for most months but rose to values exceeding 120 days during the latter months of the typical interbreeding season—September, October and November, with females exhibiting advanced ages into December (Fig. 2), and low numbers of emerging females during these months (Supplementary Appendix S4). Ninety-two flies were caught between September and December (inclusive), and the median estimated age of these flies was 124 days, with 25% of individuals estimated to be 160 days or older. The cubic spline fit of pterin relative fluorescence was highly significant for both female and male flies (Females: *F*_9_ = 24.58, *p* < 0.001, with the fit explaining 49.5% of the variance; Males: *F*_9_ = 10.87, *p* < 0.001, with the fit explaining 43.3% of the variance) indicating a strong seasonal effect. In most years, the duration of the host interbreeding period is about 210 days, and 5% of females and 3% of males that we captured exceeded this number of days (Fig. 3).

### Reproductive and mating status of wild female P. downsi

**Figure 4:**
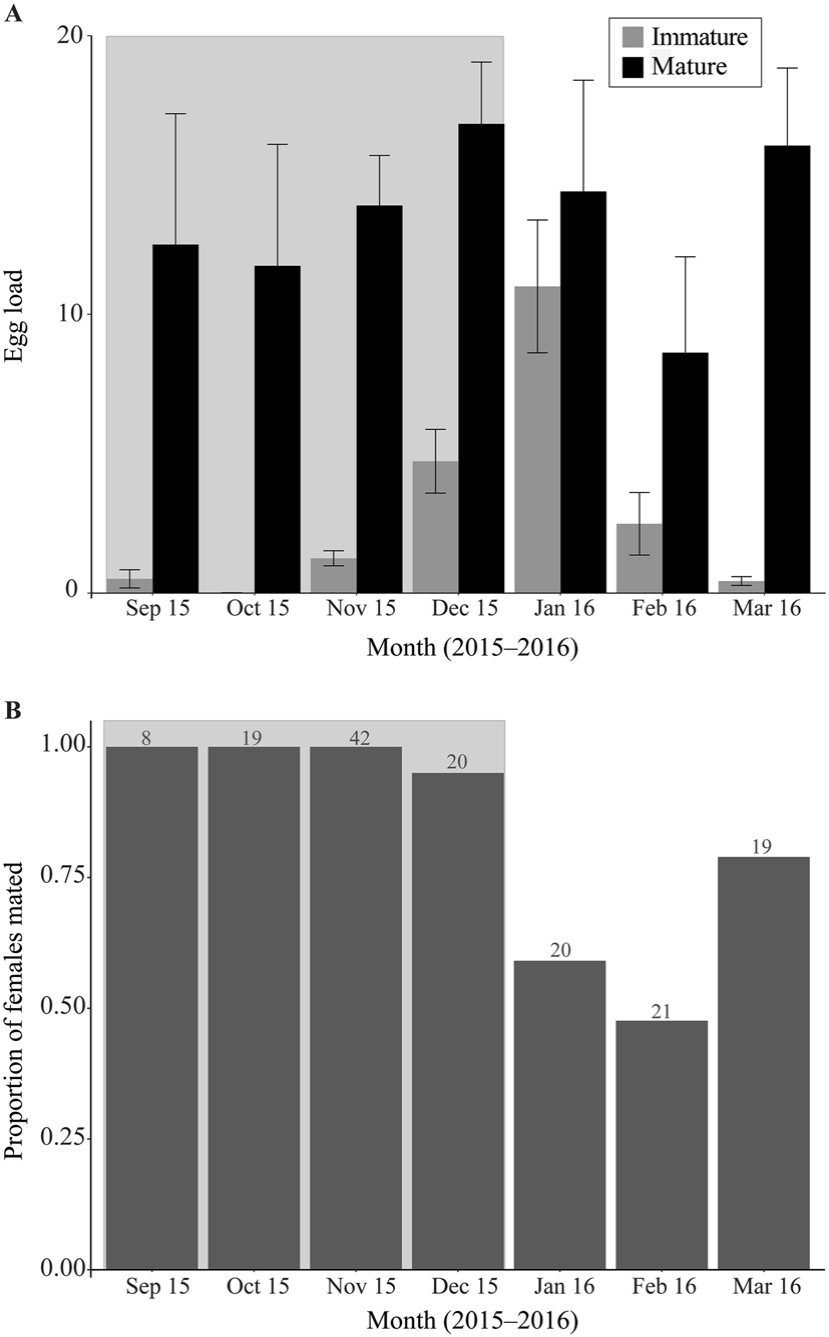
(**A**) The number of mature and immature eggs found from dissections of *P. downsi* females captured between September 2015 and March 2016 (means ± SE). (**B**) The proportion of *P. downsi* females captured between September 2015 and March 2016 that were found to contain sperm in at least one spermatheca. The grey box indicates the months of the host interbreeding period for both panels.

We dissected the abdomens of 149 *P. downsi* females captured from September 2015 through March 2016. The number of mature eggs in these flies ranged from 0 to 62 with an average of 13.51 ± 1.18, and the immature eggs ranged from 0 to 30 with an average of 2.97 ± 0.51. Head width was significantly correlated with egg load (Supplementary Appendix S5) and was therefore included as a covariate in subsequent analyses involving egg load. Average mature egg loads were remarkably consistent throughout the year (Fig. 4A) and there was no significant effect of either date or date^2^ on the mature egg load (Table 1) indicating no strong seasonal effect. The number of immature eggs showed more variability however. Female *P. downsi* carried almost no immature eggs during September, October or November, but more from December to March, leading to a significant effect of date and date^2^ on the immature egg load (Fig. 4A, Table 1). And while there was no significant relationship between mature egg load and pterin relative fluorescence (*F*_1,147_ = 0.20; *p* = 0.65), younger *P. downsi* carried significantly more immature eggs than did older *P. downsi* (*F*_1,147_ = 4.68; *p* = 0.032; immature eggs = 12.21–0.034*Relative fluorescence/1000; *r*^2^ = 0.031). Thus, the increase in immature eggs in December, which coincided with the presence of younger females (Fig. 2), likely represents new egg maturation by young flies.

**Table 1:** Results of second-order polynomial regression for effects of Date (number of days since January 1), Date^2^ and Head width on the numbers of immature and immature egg loads in field-captured *P. downsi* females.

Variable	Coefficient	Standard error	*z*	*p*
**Mature eggs**				
Intercept	− 2.125	0.4525	− 4.696	< 0.0001
Date	− 5.613e^−4^	1.341e^−3^	− 0.415	0.678
Date^2^	2.736e^−6^	3.722e^−6^	0.735	0.462
Head width	1.519	0.1409	10.78	< 0.001
**Immature eggs**				
Intercept	− 2.567	1.116	− 2.301	0.0214
Date	− 7.034e^−2^	4.336e^−3^	− 16.22	< 0.0001
Date^2^	1.847e^−4^	1.185e^−5^	15.593	< 0.0001
Head width	2.077	0.3435	6.048	< 0.0001

Of the 149 field-captured females in our study, 124 (84%) were mated, including all captured during September, October and November (Fig. 4B). The logistic regression for mating probability showed an increase with date (i.e. from January 1), but not date^2^, indicating a decline in mating levels in the host breeding period (Table 2, Fig. 4A). Lastly, we found that older females were significantly more likely to be mated than were younger females (logistic regression: *Z*_1,47_ = 4.755; *p* < 0.0001; Supplementary Appendix S6).

**Table 2:** Results of polynomial regression assessing the effect of Date (number of days since January 1), Date^2^ and Head width as a covariate on the probability of being mated for 149 *P. downsi* females captured between September 2015 and March 2016.

Variable	Coefficient	Standard error	*z*	*p*
Intercept	− 9.752	4.674	− 2.086	0.0369
Date	4.081e^−2^	1.974e^−2^	2.067	0.0388
Date^2^	− 7.335e^−5^	5.579e^−5^	− 1.315	0.1886
Head width	2.800	1.733	1.965	0.0494

## Discussion

Our goal was to evaluate three hypotheses for the persistence of the invasive fly *Philornis downsi* over the lengthy host interbreeding period in the Galapagos Islands. The absence of diapausing pupae within the nests of host birds led to the rejection of the first hypothesis—pupal diapause during the host interbreeding period. To address the second hypothesis—adult persistence over the host interbreeding period—we used an age-grading technique based on the accumulation of pteridine pigments in fly heads to demonstrate that adult *P. downsi* tend to be younger during the host breeding season than during the host interbreeding period. Estimated ages of flies also indicated that adult *P. downsi* females were capable of surviving for more than seven months, which is the length of a typical host interbreeding period. The second hypothesis was therefore supported, and it was also reinforced by data on the egg load and mating status of flies, which suggested little egg maturation or mating during the host interbreeding period. However, the estimated ages and reproductive status of some flies also provide support for the third hypothesis—that some level of reproduction occurs during the interbreeding period. Next, we discuss the potential ecological mechanisms and the implications of such a mixed strategy.

### Persistence of *P. downsi* adults during the host interbreeding period

In seasonal tropical environments, bird breeding is closely tied to the availability of arthropod prey to feed nestlings, which is linked to plant productivity and thus rain. In Galapagos lowland habitats, rain typically begins in January and continues through April or May^34,41,42^ with insect activity strongly linked to rainfall^26,43^. Nesting behaviour for most land birds begins with the onset of rain and can continue for approximately one month after it ceases^44–46^. However, rains are highly variable in the Galapagos lowlands, and bird nesting tends to be opportunistic with increased breeding coinciding with sufficient rain at any time of the year^42,47^. Our study period included 2015, which has been classified as an El Niño year^35,36^, and was thus characterized by higher rainfall than usual. Given the precipitation patterns at our field site and several cases of finches breeding in June and July^48^, we conclude that the nesting season was increased by 4–6 weeks during 2015.

The oldest female flies were captured during the months of September, October, November and December. In a typical year, *P. downsi* adults would have to reach ages exceeding seven months (ca. 210 days) to span the time from one bird breeding season to the next. While this threshold was up to 42 days shorter due to El Niño conditions (see above), the estimated age of 5% of female *P. downsi* exceeded seven months, showing a capacity to survive more typical host interbreeding periods. Female trap catches stayed relatively high and stable throughout the host interbreeding period of our study, but males almost disappeared from traps entirely during September, October and November, similar to findings in 2012–2014^28^. The lower trap catch of males during the interbreeding season is consistent with shorter longevity and limited recruitment of males during this period^28^.

Taken together, our data provide strong evidence that adult *P. downsi* females—and to a much lesser extent, males—are able to persist through the interbreeding season in the Galapagos lowlands. This ability undoubtedly aided the establishment of these flies upon their invasion from mainland South America^21^. Exactly how they survive the entire host interbreeding season is not clear however, since laboratory rearing has shown that moisture and a sugar-rich diet are needed for survival of adult *P. downsi*. It is most likely that flies feed from flowers and fruits, and while most plants in Galapagos flower during the rainy season, some do produce flowers and fruits during the dry season^49^. Traveset et al.^50^ reported nine plant species that flowered in the lowlands during the interbreeding season and observed *P. downsi* feeding on flowers of the snakeweed *Stachytarpheta cayennensis* in September in the lowlands of San Cristobal Island. There is also a report of *P. downsi* feeding on fruits of the shrub *Tournefortia pubescens* in the lowlands of Santa Cruz during the host interbreeding period. Floral and fruit resources for adult *P. downsi* require more study.

### *Philornis downsi* reproduction during host interbreeding periods

A quarter of females analysed during the last three months of the interbreeding season were estimated to be fewer than 92 days old, suggesting that some fly reproduction did occur. Below, we consider three non-exclusive hypotheses that could explain this limited level of recruitment during the host interbreeding period.

#### Hypothesis 1

*Philornis downsi* reproduce during the host interbreeding period in the Galapagos lowlands.

Limited nesting by some bird species was recorded in the lowlands during June and July of 2015. This was likely related to high precipitation in May and is indicative of opportunistic nesting^42,47^. Darwin’s finches typically breed in the hot season^42,47^, but little is known about the breeding habits of eight other bird species that are known to be hosts of *P. downsi* in the lowlands of Santa Cruz (Supplementary Appendix S7). There are limited records of cool-season nesting for two of these species. The Galapagos Flycatcher, *Myiarchus magnirostrus*, nests in cavities and is known to breed during the hot season^48,51^. However, an active flycatcher nest was discovered with nestlings and *P. downsi* at the CDRS during August 2014^52^ as well as in June of 2015 at our field site^48^. Another possibility is the Galapagos Dove, *Zenaida galapagoensis*. Some active but unparasitized dove nests were reported during the interbreeding season on Daphne Major and Baltra Islands^24^. No records of cool-season nesting have been reported in the lowlands for any of the remaining six non-finch hosts of *P. downsi* (Supplementary Appendix S7) but this lack of observations does not rule out such activity.

#### Hypothesis 2

*Philornis downsi* reproduce during the host interbreeding period by parasitizing birds in the Galapagos highlands.

In the mesic highlands, precipitation is more evenly spaced throughout the year^34^, and this can lead to earlier nesting by some finch species^41,42^. Occasional finch nests (all containing *P. downsi*) were reported during the interbreeding seasons of 2012–2014^28^. For highland reproduction to explain our finding of young *P. downsi* females captured in the lowlands at the end of the rainy season, the flies would need to travel from the highlands to our lowland trapping sites, a distance of approximately 5 km. *Philornis downsi* could conceivably migrate between the Santa Cruz highlands and lowlands, either on a daily or seasonal basis, as a previous study documented no genetic structure according to habitat type, which implies some dispersal between the two habitats^53^. So far though, this hypothesis rests on two untested assumptions: year-round breeding in the highlands by the flies and annual or daily dispersal to the lowlands.

#### Hypothesis 3

*Philornis downsi* reproduce during the host interbreeding period in lowland habitats by feeding on adult birds.

Recent studies have confirmed that *P. downsi* larvae are capable of feeding on adult birds while they are incubating eggs^21,54,55^. This phenomenon may be linked to selection for early reproduction by *P. downsi* under conditions of intense intraspecific competition in Galapagos^56^. If such behaviour were extended to birds consistently roosting in recently used nests for shelter, it could provide the flies a means of reproducing during the interbreeding season. However, we are not aware of any known hosts of *P. downsi* in Galapagos roosting in nests during the interbreeding period.

### Egg maturation and mating

The number of mature eggs carried by field-captured *P. downsi* females differed little between seasons, as previously reported^28^. During the bird nesting season, this probably reflects a pattern of ovarian homeostasis in which reductions in egg load through oviposition stimulate egg maturation^57,58^. During the interbreeding season, however, the constancy of mature egg loads more likely reflects a cessation of both oviposition and egg maturation, as well as an inability to resorb mature eggs, which are surrounded by a thick and complex plastron^38^. Resorption of immature eggs, however, is known from many dipterans, e.g.^58–61^ and may be responsible for the very low numbers of immature eggs found in females captured during the interbreeding season. Nutrients recycled through resorption of immature eggs could help *P. downsi* survive through the late interbreeding season in Galapagos, which is also a time when nutritional resources for adults such as ripe fruit are limited, particularly in the lowlands^49^.

Our spermathecal dissections indicated that females tend to mate during the host breeding season and store sperm during the host interbreeding season, and the number of males found during the interbreeding season was very low, as found previously^28^. Thus, females mate during the nesting season and retain sperm over the interbreeding season.

### A hypothesis to explain the absence of pupal diapause

Despite the presence of limited reproductive opportunities during the host interbreeding season in the lowlands, host availability is much greater during the hot season^26,62^. Also, mortality of adult *P. downsi* is presumably high over the seven or so months of the host interbreeding period. We thus view the absence of developmental arrest during the host interbreeding period in *P. downsi* as paradoxical. Other parasites of seasonally breeding birds have evolved specialized synchronization mechanisms such as pupal diapause^13,63,64^ but such strategies appear to be absent in Galapagos *P. downsi*. We hypothesize that diapause is not observed because of a legacy of gene flow between areas with seasonal and continuous host breeding patterns in mainland South America, the native range of *P. downsi*. *Philornis downsi* is a generalist parasite of birds and is thus presumably able to parasitize hosts throughout the year in areas that experience year-round rainfall^65^, although a recent study in Galapagos found selection pressures acting on larval survival that may result in *P. downsi* shifting towards host specialization^66^. Within its native geographic range, *P. downsi* has been reared from the nests of multiple bird species throughout the year on Trinidad^15^ and Brazil^67^, both characterized by humid conditions that support bird nesting throughout the year. Under these circumstances, adaptations like pupal diapause that restrict reproduction would likely be maladaptive. Other locations within the known native range of *P. downsi* exhibit seasonal rains with a strong dry season during which few land birds nest such as Western mainland Ecuador^19^ and various sites in Southern Brazil and Northern Argentina^16–18,68^. Although seasonal pupal diapause in these subtropical localities may be adaptive, *P. downsi* appears to exhibit high vagility^21^ and gene flow among populations can be high^53,69^, decreasing the opportunity for local adaptation^70^.

## Conclusions

The year-round persistence of the invasive avian vampire fly, *Philornis downsi*, in the Galapagos Islands has been a mystery since its bird hosts are largely unavailable during a 7-month interbreeding period. We used a pterin-based age-grading technique along with dissections of adult flies and puparia to test various hypotheses that could explain this persistence. The results suggest that the flies do not enter diapause during the host interbreeding period but rather use a mixed strategy of adult female survival and the use of rare cases of birds breeding during the interbreeding period to persist. We discuss possible mechanisms for both survival and bird parasitism during the host interbreeding period and propose a hypothesis for the paradoxical lack of diapause in *P. downsi* in the Galapagos Islands.

## Supplementary Information

Supplementary Information.

## Data Availability

Data used in this study have been made available at the Data Repository for the University of Minnesota (DRUM): https://doi.org/10.13020/7zyy-0125.
