# Supplementary material for: Persistence of the invasive bird-parasitic fly Philornis downsi over the host interbreeding period in the Galapagos Islands
Source: Sci Rep. 2022 Feb 11;12:2325. doi: 10.1038/s41598-022-06208-5 (PMC8837626; doi:10.1038/s41598-022-06208-5)
Supplement: Supplementary file 1 — Supplementary Information. [file 41598_2022_6208_MOESM1_ESM.pdf]

Supplementary Information for

Persistence of the invasive bird-parasitic fly *Philornis downsi* over the host interbreeding period in the Galapagos Islands

Mariana Bulgarella[1,*] M. Piedad Lincango[2,3], Paola F. Lahuatte[2], Jonathan D. Oliver[1], Andrea Cahuana[2], Ismael E. Ramírez[1], Roxanne Sage[1], Alyssa J. Colwitz[4], Deborah A. Freund[4], James R. Miksanek[1], Roger D. Moon[1], Charlotte E. Causton[2] and George E. Heimpel[1]

[1]Department of Entomology, University of Minnesota, St. Paul, Minnesota, United States of America

*Current address: School of Biological Sciences, Victoria University of Wellington, Wellington, New Zealand

[2]Charles Darwin Research Station, Charles Darwin Foundation, Puerto Ayora, Santa Cruz Island, Galapagos Islands, Ecuador

[3]Facultad de Ciencias Agrícolas, Universidad Central del Ecuador, Quito, Ecuador

[4]Biology Department, University of Wisconsin Eau Claire, Eau Claire, Wisconsin, United States of America

This PDF file includes:

Appendices S1 to S7

SI References

Appendix S1: **Age calibration under laboratory versus field conditions**

Performing age calibration curves under laboratory conditions is preferable to performing them under field conditions because of the ability to control conditions and (often) obtain flies with a broader range of ages. However, some studies have shown that pteridines can accumulate at different rates under field vs. laboratory conditions, because of variation in ambient temperature and ability of flies to thermoregulate through choice of perching sites[1]. Thus, it is important to determine whether age calibration curves differ under these conditions so that correction factors can be developed, or methods modified if needed. In order to test for an effect of rearing conditions on pterin accumulation we reared flies from puparia collected from wild bird nests as described in the main text and assigned them randomly to a laboratory or a field group. Within 1 h of emergence, flies were placed in groups into one of two 1 x 1 x 1 m wood/plexiglass/mesh cages that were held either inside the laboratory at the Charles Darwin Research Station (CDRS) on Santa Cruz Island, Galapagos, Ecuador (see main text) or outside protected from the direct sun. Both groups were supplied with a papaya diet (see main text) and water *ad libitum*. A total of 20 flies were laboratory reared (n = 14 females; n = 6 males) and a total of 18 flies were field reared (n = 13 females; n = 5 males). Flies were maintained in each group for a total of 42 days. Flies from each group, chosen at random, were removed for pterin measurement at 3, 7, 14, 21, 28, 35, and 42 days. Heads were removed and stored until shipment to the University of Minnesota. Head width measurements, sex determination (Fig. S1) and the quantification of pterin were done as described in the main text.

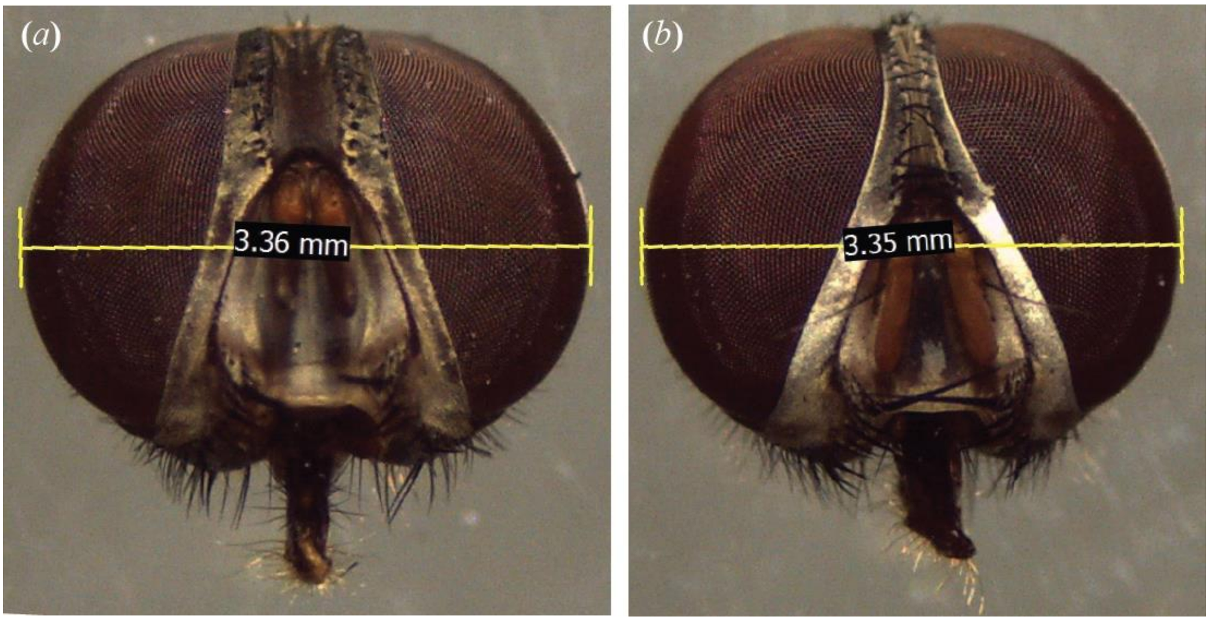

Figure S1. Photographs of *P. downsi* heads showing size measurement of (A) a female and (B) a male fly. Male eyes meet in the middle, while female eyes are farther apart. Photo credits: M. Bulgarella.

Males and females did not differ significantly with respect to relative fluorescence (t = 1.92; p = 0.07) and so were pooled for these analyses. Also – there was no significant effect of *P. downsi* head width on relative fluorescence ($F_{1, 36}$ = 1.98, p = 0.168) so relative fluorescence values uncorrected for head size were used. We used an analysis of covariance to compare the slopes of relative fluorescence (estimated as in the main text) on age of male and female *P. downsi* pooled in the two treatments (laboratory vs. field conditions). This showed a strong effect of *P. downsi* age but no significant effect of either treatment or the interaction between age and treatment (Table S1, Fig. S2). This analysis shows that our laboratory conditions provide a statistically indistinguishable age calibration as do field-cage conditions, justifying the use of a laboratory-based age calibration curve for the main study.

Table S1. Analysis of covariance for effects of rearing environment (laboratory or field cages), age of *P. downsi* in days, and the interaction of these two variables on pterin relative fluorescence.

**Source**	**df**	***F* value**	***p***
**Environment**	1	0.18	0.671
**Age**	1	62.44	<0.001
**Environment*Age**	1	0.16	0.694

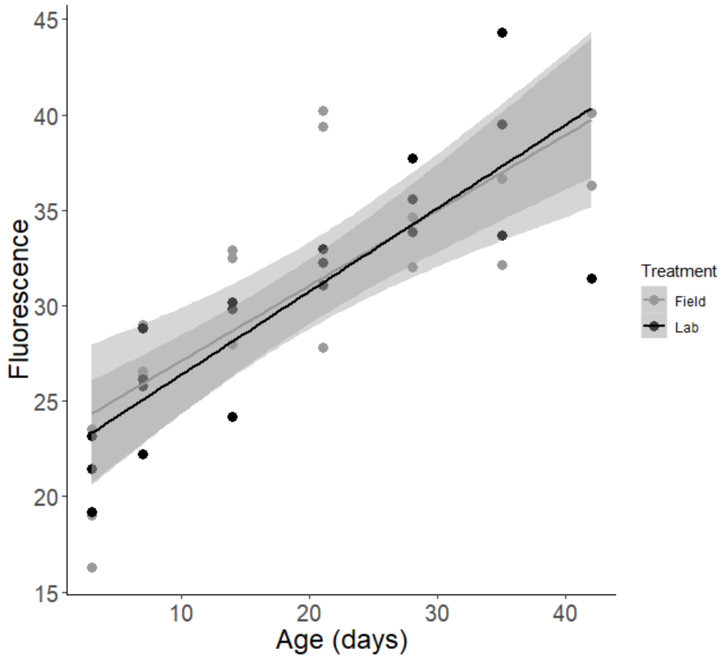

Figure S2. Pterin relative fluorescence (in thousands) from *P. downsi* adults (females and m ales pooled) of known ages reared under laboratory and field-cage conditions with 95% confi dence interval bands on the regression models as calculated using the function *geom_smooth* within the R package *ggplot2*[2].

Appendix S2: **Patterns of precipitation at field site**

To characterize recent precipitation patterns at our field site, we calculated weekly precipitation totals as recorded at the CDRS at Academy Bay, Puerto Ayora, Santa Cruz Island (0° 44'37.06"S, 90° 18'7.94"W, elevation: 2 m asl) from 1 Jan, 2012 through 31 Dec, 2016. The weather station is approximately 1 km from the El Barranco site and the data spanned three years before and one year after our field study began and included the study interval reported by Causton et al.[3].

Precipitation rates were higher than usual at our site in April, May, November and December of 2015 (Fig. S3; see also[4]), indicating that the time of year traditionally considered to be the dry season was shorter by approximately two months and lasted from June through October in 2015. The 2015/2016 season was considered an El Niño year, which is typically characterized by increased precipitation[4,5].

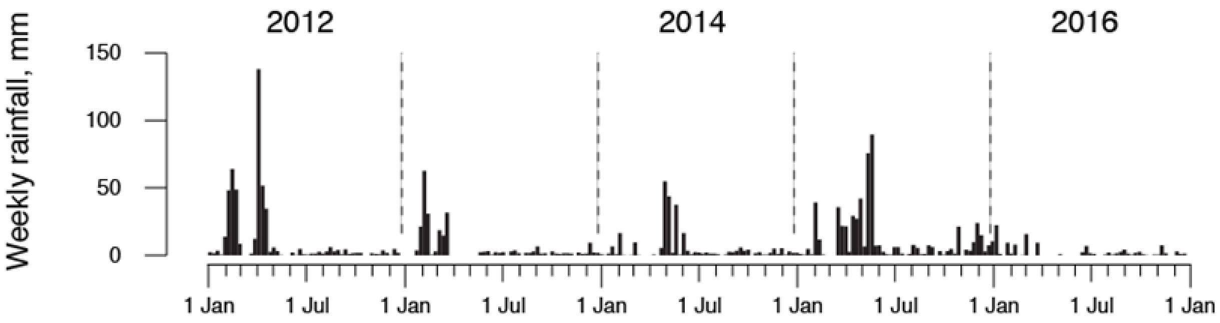

Figure S3. Weekly precipitation recorded at the Charles Darwin Research Station in the lowland region of Santa Cruz Island, Galapagos, 2012–2016, inclusive.

Appendix S3: Incorporation of head width and non-linear fitting into the calibration process

The GLM including sex, age, head width as well as the interaction between sex and age indicated that all of these variables had a significant effect on pterin relative fluorescence (Table S2).

Table S2. Regression coefficients estimated with a Generalized Linear model with gamma error distribution testing for the effects of age, sex, and head width, and the interaction between age and sex on pterin relative fluorescence levels (*RF*, in thousands). Null deviance was 18.2 with 67 degrees of freedom.

**Parameter**	**Coefficient**	**Standard Error**	***t***	***p***
**Intercept**	- 17.260	6.872	-2.52	0.015
**Age, in days**	0.310	0.028	11.1	< 0.001
**Sex (**M vs. F**)**	6.706	2.412	2.78	0.008
**Head width**	10.477	2.357	4.45	<0.001
**Age*Sex**	-0.198	0.040	-4.89	<0.001

Since relative fluorescence (*RF*) increased significantly with the head width (*H*) of *P. downsi* adults in the complete generalized linear model (Table S2), we reasoned that composite variables in which relative fluorescence is corrected for head width may provide a better fit to age and thus a more accurate calibration. We tested for the effect of three such composite variables on the age of female and male *P. downsi*: RF/H, RF/H^2 and RF/H^3 using linear regression. Resulting r^2 values were compared to the linear regression using only relative fluorescence (see Fig. 1 in main text). While regressions of the composite variables on age produced slightly higher r^2 values than only relative fluorescence for females, the fit for all

composite variables was worse for males than only relative fluorescence (Table S3). We therefore decided that adjustment for head width was unwarranted.

Table S3. Coefficient of determination (r^2) for linear regressions between four dependent variables and the known age of laboratory-held *P. downsi* female or male adults. The first model (*RF* ~ Age) is shown in Fig. 1 of the main text and the other three models involve composite dependent variables.

Model	r^2 for Females	r^2 for Males
***RF* ~ Age**	0.741	0.671
***RF/H* ~ Age**	0.776	0.624
**RF/H^2 ~ Age**	0.784	0.503
**RF/H^3 ~ Age**	0.763	0.313

Visual inspection of the calibration curve of pterin fluorescence on known age suggest a possible non-linear relationship (see Fig. 1 of the main text). We therefore investigated three non-linear functions linking fluorescence to age for females and males separately. Some other pterin age-grading studies have used non-linear relationships to estimate age from relative fluorescence[6]. We tested a second-order polynomial (quadratic) and two asymptotic relationships. The potential usefulness of these relationships is discussed below.

Quadratic Fit. While the quadratic fit produced a fit of relative fluorescence to age with an coefficient of determination (adjusted r^2; 0.825) that is higher than that of the linear fit (0.746) with significant effects of both the Age and the Age^2 terms for female *P. downsi*, the same cannot be said for males, for which neither term was significant and the adjusted r^2 value (0.662) was slightly lower than that produced by the linear fit of 0.674 (Fig. S4, Table S4).

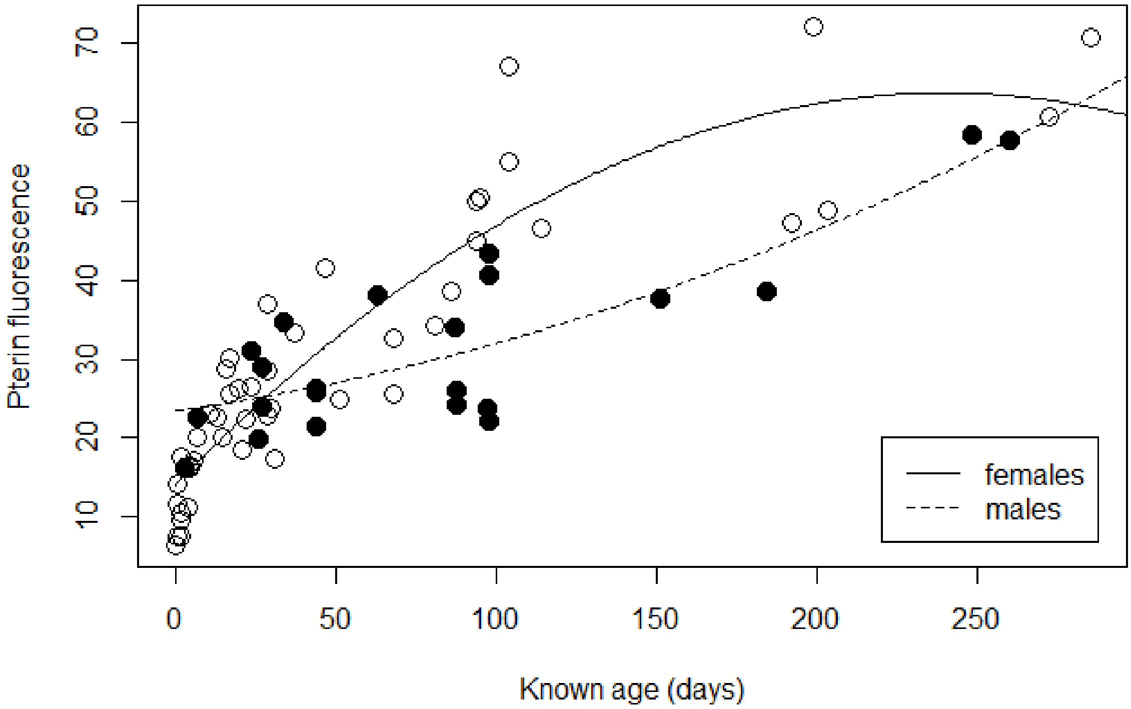

Figure S4. Pterin relative fluorescence for *P. downsi* females (open circles) and males (closed circles) of known ages along with the fits of these data to second-order polynomial (quadratic) models separately for females and males.

Table S4. Results of linear model analysis for quadratic fits of the form relative fluorescence = a*Age + b*Age^2 + c for female and male *P. downsi* in the calibration study.

**Parameter**	**Coefficient**	**St. Error**	***t***	***p***
		**Females**		
**Intercept (*c*)**	14.0193102	1.6503525	8.495	9.55e-11
b	0.4148401	0.0461059	8.998	1.93e-11
a	-0.0008658	0.0001834	-4.721	2.51e-05
		**Males**		
**Intercept (*c*)**	2.343e+01	3.259e+00	7.189	7.89e-07
b	5.635e-02	6.857e-02	0.822	0.421
a	2.910e-04	2.588e-04	1.125	0.275

In addition, the maximum level of fluorescence recorded from field-caught *P. downsi* exceeds the maximum level recorded in the calibration study, making the age estimates for these female individuals uninterpretable using the quadratic calibration equation. Given

these considerations, a quadratic fit was rejected for predicting age from relative fluorescence data of field-caught *P. downsi*.

Asymptotic Fit 1: Michaelis-Menten form. The first of the asymptotic fits was of the general Michaelis-Menten form, *RF* = *a**Age/(*b* + Age), where *RF* is pterin relative fluorescence, *a* is an estimate of maximum (asymptotic) relative fluorescence and *b* is an estimate of the age corresponding to half of the saturation level of relative fluorescence. An estimate of the proportion of variance explained by the model was made by calculating the sum square total (sst) and sum of squares error (sse) directly from the data to produce an estimate of r^2 ([sst – sse]/sst)[7]. This estimate of r^2 was slightly higher for the Michaelis-Menten model (0.755) than for the linear model (0.746) for female *P. downsi* but much lower for males (0.340 vs. 0.674) (see Fig. S5). Estimates for *a* were significant for both female and male flies, but *b* was only significant for females (Table S5). For these reasons we did not consider this model to provide a superior fit to the data than the linear model.

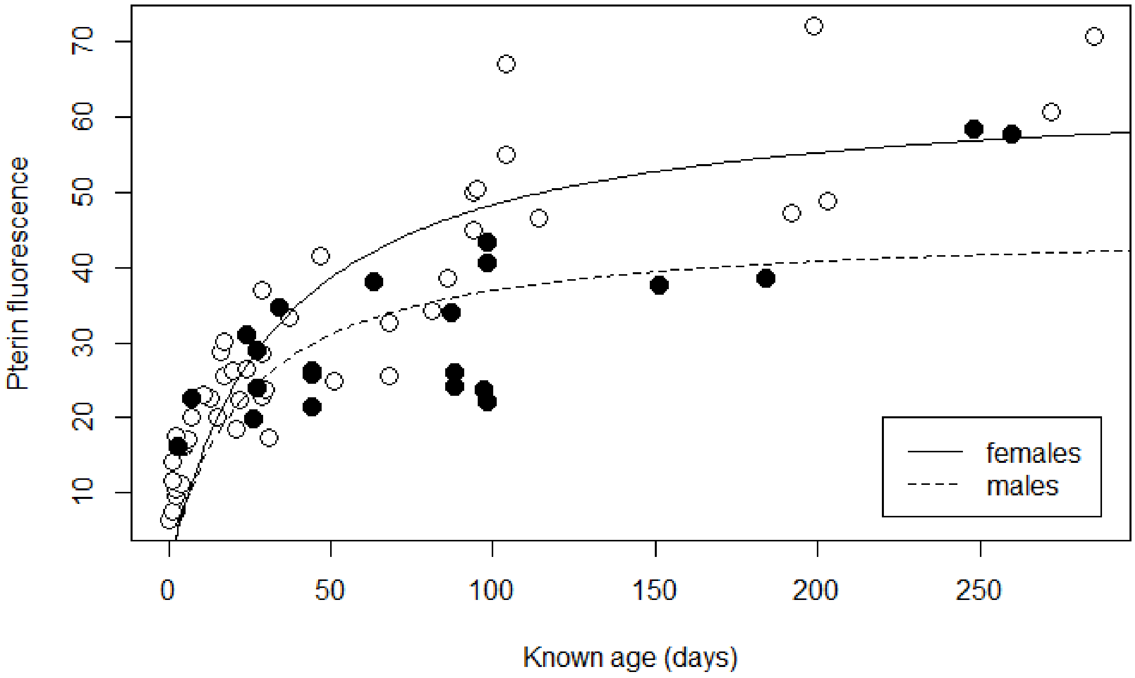

Figure S5. Pterin relative fluorescence for *P. downsi* females (open circles) and males (closed circles) of known ages along with the fits of these data to Michaelis-Menten models separately for females and males.

Table S5. Results of non-linear fitting analysis for Michaelis-Menten models of the form Relative fluorescence = a*Age/(b + Age) for female and male *P. downsi* in the calibration study.

Parameter	Coefficient	St. Error	*t*	*p*
		**Females**		
a	64.505	4.928	13.088	< 2e-16
b	33.559	7.322	4.583	3.773e-05
		**Males**		
a	45.564	5.915	7.703	2.08e-07
b	23.419	11.596	2.020	0.057

Asymptotic Fit 2: Power Function. The second asymptotic fit was a power function of the form $RF = a*Age^k$ of which a and k are fitted parameters. This model was used in age calibration of the tephritid fruit fly *Anastrepha ludens* by Tomic-Carruthers et al.[6]. For our data set, these fits produced high estimated r^2 levels (0.844 and 0.566 for females and males, respectively) using the same method for estimating r^2 as for the Michaelis-Menten form, and both parameters were significant for males and females (Fig. S6, Table S6). However, the age estimates from field-caught flies using this model included values that we deemed to be unrealistically high, exceeding 550 days for females and 650 days for males, respectively, and we rejected this model for that reason. Any model with a horizontal asymptote, including both the power and Michaelis-Menton models, will have limited utility in predicting x-values (age) for high values of y (relative fluorescence). This is because variation in relative fluorescence among older-aged flies of unknown age will be disproportionately displaced along the saturating curve of mean estimated values (model fit) when making inverse predictions.

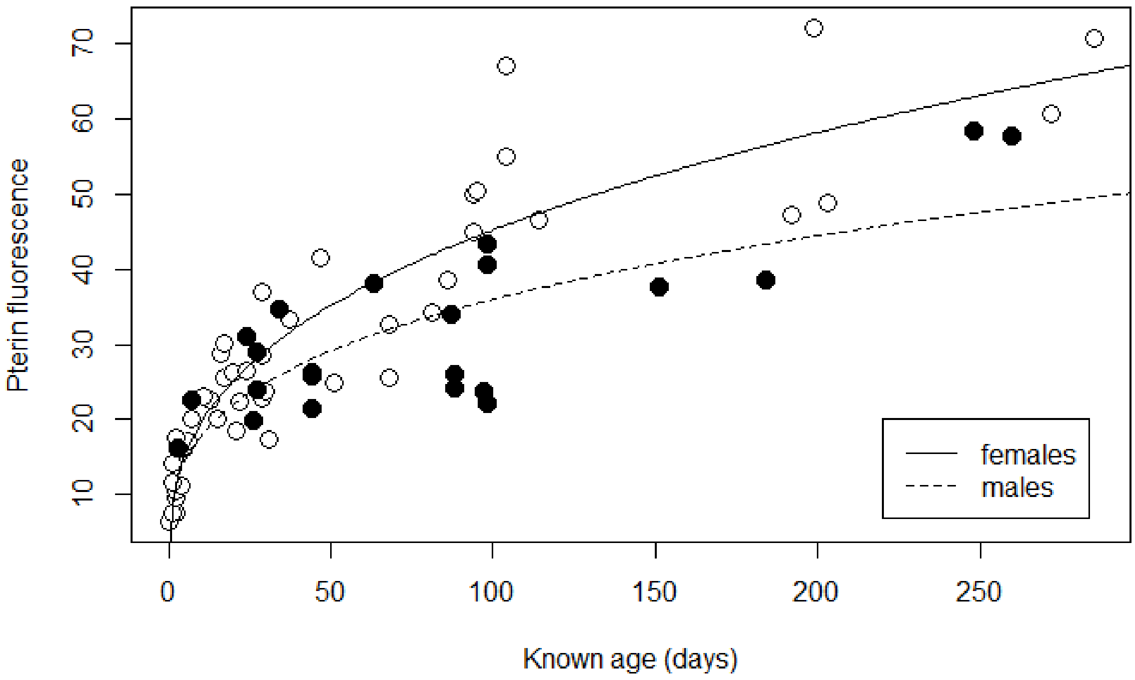

Figure S6. Pterin relative fluorescence for *P. downsi* females (open circles) and males (closed circles) of known ages along with the fits of these data to the monomial power models separately for females and males.

Table S6. Results of non-linear fitting analysis for the monomial power models of the form Relative fluorescence = a*Age^k for female and male *P. downsi* in the calibration study.

Parameter	Coefficient	St. Error	*t*	*p*
		**Females**		
a	8.46473	1.08058	7.834	7e-10
k	0.36377	0.02811	12.939	<2e-16
		**Males**		
a	8.81399	2.50432	3.520	0.00216
k	0.30539	0.06181	4.941	7.88e-05

Appendix S4: Estimated month of eclosion for field-captured *P. downsi* adults

We estimated the month of eclosion for field-captured *P. downsi* adults based upon the estimated ages shown in Figure 1 of the main text and the date of capture (Fig. S7).

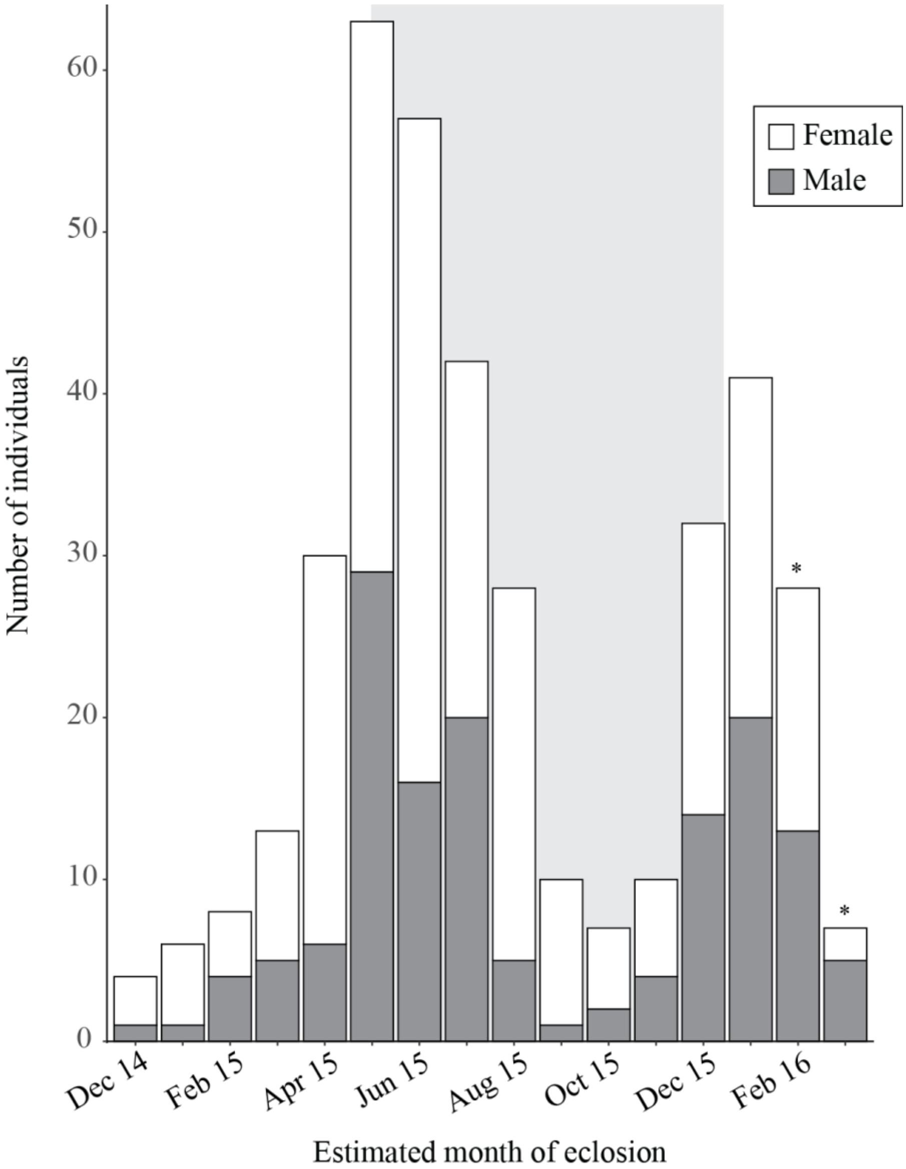

Figure S7. Estimated month of eclosion for field-captured *P. downsi* adults. Estimated ages below zero days are standardized to zero for the calculations. The grey box indicates months that are typically dry in the lowlands of Galapagos and do not support nesting of most *P. downsi* hosts. *Values are likely substantially underestimated during these months because sampling ended on 17 March, 2016.

Appendix S5: Effect of head width on the sum of mature and immature egg loads for field-collected *P. downsi*

Head width had a significant but weak effect on both mature and immature egg loads (Generalized Linear model with Negative Binomial error distribution and log link: $z = 3.064$, $p = 0.002$; residual deviance was 175.4 on 147 d.f.). See Fig. S8.

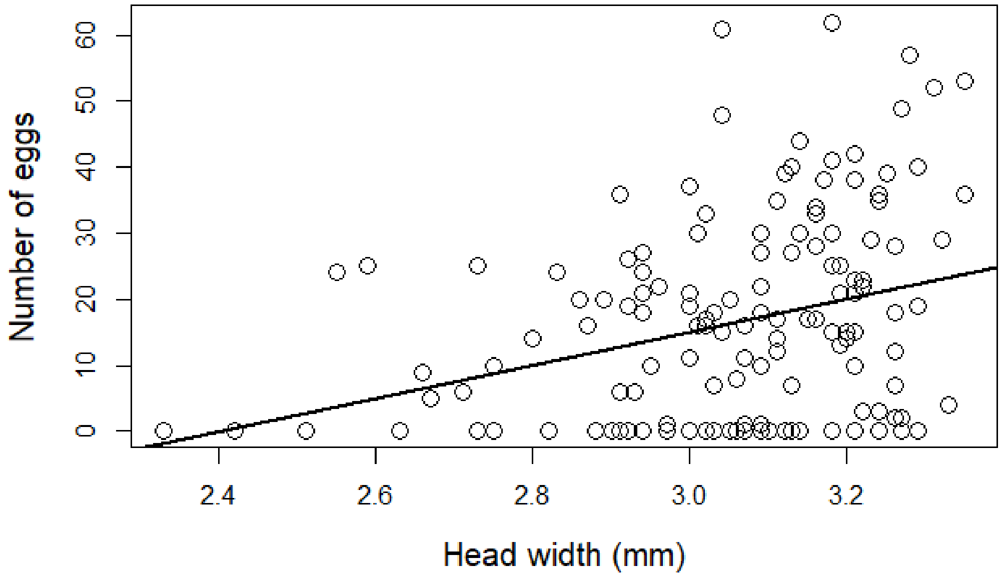

Figure S8. The effect of *P. downsi* head width (in mm) on the sum of mature and immature eggs in field-captured *P. downsi* females. The line is from a simple linear regression: Eggs = Head*25.099 – 60.276; $r^2 = 0.098$.

Appendix S6: Probability of being mated as a function of age for field-caught female *P. downsi*

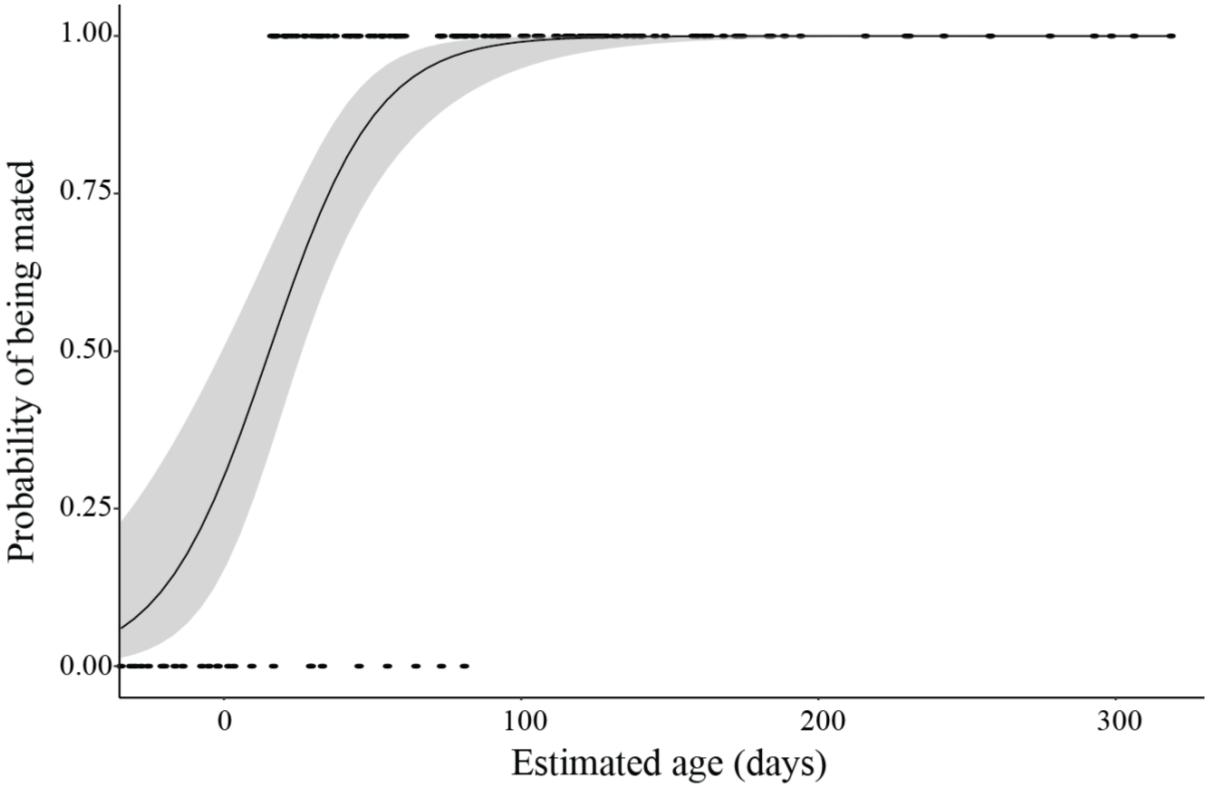

Figure S9. The probability of being mated as a function of age for field-caught female flies. The points indicate individual females for which all three spermathecae were empty (0) or for which at least one spermatheca contained sperm (1). Logistic regression: $Z_{1,47} = 4.755$; $p < 0.0001$. The line is a logistic regression fit for probability of being mated = 1/(1 + exp(-(0.0552 *Age - 0.8418))). See main text for statistical analysis.

Appendix S7: Breeding characteristics of eight non-finch bird species resident in Santa Cruz Island that are known hosts of *P. downsi*

Table S7. Breeding characteristics of eight non-finch bird species resident in Santa Cruz Island that are known hosts of *P. downsi.* Noted is whether bird species are mainly found in lowland or highland habitats, and whether there is evidence of breeding during the host interbreeding season, typically considered not to support bird reproduction.

**Species**	Mainly found in **lowlands** or **highlands**	**Evidence of breeding in cool season**	**References**
Galapagos Dove (*Zenaida galapagoensis*)	Lowlands	**Yes**	8
Dark-billed Cuckoo (*Coccyzus melacoryphus*)	Lowlands	No	9,10
Smooth-billed Ani (*Crotophaga ani*)	Both	No	10,11
Galapagos Martin (*Progne modesta*)	Both	No	12
Galapagos Mockingbird (*Mimus parvulus*)	Lowlands	No	10,13
Yellow Warbler (*Setophaga petechia*)	Both	No	10,14
Galapagos Flycatcher (*Myiarchus magnirostrus*)	Lowlands	**Yes**	15,16
Little Vermillion Flycatcher (*Pyrocephalus nanus*)	Both	No	10,17

Supplementary Information References

1. Hayes, E. J. & Wall, R. Age-grading adult insects: a review of techniques. *Physiol. Entomol.* **24**, 1–10 (1999).
2. Wickham, H. ggplot2: Elegant Graphics for Data Analysis (Springer-Verlag, New York, USA, 2016).
3. Causton, C. E., Moon, R. D., Cimadom, A., Boulton, R. A., Cedeño, D., Lincango, M. P., Tebbich, S. & Ulloa, A. Population dynamics of an invasive bird parasite, *Philornis downsi* (Diptera: Muscidae), in the Galapagos Islands. PLoS ONE 14(10): e0224125, https://doi.org/10.1371/journal.pone.0224125 (2019).
4. Martin, N. J., Conroy, J. L., Noone, D., Cobb, K. M., Konecky, B. L. & Rea, S. Seasonal and ENSO influences on the stable isotopic composition of Galapagos precipitation. *J. Geophys. Res-Atmos.* **123**, 261–275, https://doi.org/10.1002/2017JD027380 (2018).
5. Stramma, L., Fischer, T., Grundle, D. S., Krahmann, G., Bange, H. W. & Marandino, C. A. Observed El Niño conditions in the eastern tropical Pacific in October 2015. *Ocean Sci.* **12**, 861–873, https://doi.org/10.5194/os-12-861-2016 (2016).
6. Tomic-Carruthers, N., Mangan, R. & Carruthers, R. Age estimation of Mexican fruit fly (Diptera: Tephritidae) based on accumulation of pterins. *J. Econ. Entomol.* **95**, 1319–1325 (2002).
7. Crawley, M. J. The R Book (Wiley, 2013).
8. Coloma, A., Anchundia, D., Piedrahita, P., Pike, C. & Fessl, B. Observations on the nesting of the Galapagos Dove, *Zenaida galapagoensis*, in Galapagos, Ecuador. *Galapagos Research* **69**, 34–38 (2020).
9. Ervin, S. The nesting of the Dark-billed Cuckoo in the Galapagos. *Noticias de Galapagos*, **48**, 8–10 (1989).

10. Fessl, B. & Tebbich, S. *Philornis downsi* - a recently discovered parasite on the Galapagos archipelago - a threat to Darwin's finches? *Ibis,* **144**, 445–451 (2002).
11. Cooke, S. C., Anchundia, D., Caton, E., Haskell, L. E., Jäger, H., Kalki, Y., Mollá, Ó., Rodríguez, J., Schramer, T. D., Walentowitz, A. & Fessl, B. Endemic species predation by the introduced smooth-billed ani in Galápagos. *Biol. Invasions.* **22**, 2113–2120 (2020).
12. Anchundia, D. & Fessl, B. The conservation status of the Galapagos Martin, *Progne modesta*: assessment of historical records and results of recent surveys. *Bird Conserv. Int.* **31**, 129–138, https://doi.org/10.1017/S095927092000009X (2021).
13. Curry, R. L. & Grant, P. R. Demography of the cooperatively breeding Galapagos mockingbird, *Nesomimus parvulus*, in a climatically variable environment. *J. Anim. Ecol.* **58**, 441–463, https://doi.org/10.2307/4841 (1989).
14. Snow, D. W. Annual cycle of yellow warbler in Galapagos. *Bird Band.* **37**, 44–49 (1966).
15. Ervin, S. Nesting behavior of the large-billed flycatcher on Isla Santa Cruz. *Noticias de Galapagos*, **51**, 17–20 (1992).
16. Lincango, P., Causton, C., Cedeno, D., Castañeda, J., Hillstrom, A. & Freund, D. Interactions between the avian parasite, *Philornis downsi* (Diptera: Muscidae) and the Galapagos Flycatcher, *Myiarchus magnirostris* Gould (Passeriformes: Tyrannidae). *J. Wildl. Dis.* **51**, 907–910, https://doi.org/10.7589/2015-01-025 (2015).
17. Mosquera, D. The effect of *Philornis downsi* in the reproductive success of the Little Vermilion Flycatcher on the Isabela Island, Galapagos. M.Sc. dissertation, University of Vienna. Vienna, Austria (2018).
